# Supplementary material for: Long-term mortality in mothers of infants with neonatal abstinence syndrome: A population-based parallel-cohort study in England and Ontario, Canada
Source: PLoS Med. 2019 Nov 26;16(11):e1002974. doi: 10.1371/journal.pmed.1002974 (PMC6879118; doi:10.1371/journal.pmed.1002974)
Supplement: S1 Table — (DOCX) [file pmed.1002974.s001.docx]

**S1 Table**. Study diagnostic codes and description of sociodemographic characteristics.

|  | ICD-10* | | ICD-9† | | DSM-IV‡ | | Procedural Codes§ |
| --- | --- | --- | --- | --- | --- | --- | --- |
| Baseline characteristics | |  | |  | |  | |
| Any psychiatric condition | ICD-10 codes listed below for: addictions-related and other mental health (excluding addictions) | | ICD-9 codes listed below for: addictions-related and other mental health (excluding addictions) | | All DSM-IV codes, excluding dementia (290.x and 294.x and provisional diagnosis 2) | | N/A |
| Addictions-related | F55, F10 to F19, G24.0, G31.2, G40.5, G62.1, G72.0, G72.1, I42.6, K29.2, K70, K85.2, K85.3, K86.0, O35.4, R78.1-R78.5, Y47, Y49, Z50.2, Z50.3, Z71.4, Z71.5, Z72.2, Z86.4; | | 291.x (0-5, 8, 9), 292.x (0, 1, 2, 8, 9), 303.x, 304.x, 305.x, 3337, 30390, 3457, 3575, 3594, 3594, 4255, 53530, 5710, 5770, 5770, 5771, 65541, 7960, 7960, 7960, 7960, 7960, E9370, E9390, V5789, V5789, V6542, V6542, V698, V1589, 5711, 5712, 5713, 5728, 65543, 65540, E9394, E9371, E9372, E9373, E9376, E9378, E9379, E9390, E9391, E9392, E9393, E9396, E9397, E9398, E9399 | | 291.x (excluding 291.82), 292.x (excluding 292.85), 303.x, 304.x, 305.x, provisional diagnosis 4 | | N/A |
| Other mental  health (excluding  addictions) | F04 to F99, Z09.3, Z50.4, Z86.5, Z91.4, X60-X84, Y10-Y19, Y28, Y87.2, Y34  Note: Excludes addictions-related codes listed above | | 290-319 (excluding 290.x, 294.x), V673, V5789, V118, V1549, E950-E958, E980, E986   Note: Excludes addictions-related codes listed above | | All DSM-IV codes, excluding dementia (290.x, 294.x, and provisional diagnosis 2) and addictions-related codes listed above | | N/A |
| Caesarean delivery | N/A | | N/A | | N/A | | Ontario: 860, 861, 862, 5MD60; England: R17, R18 |
| Pre-eclampsia / Eclampsia | O14, O15 | | N/A | | N/A | | N/A |
| Gestational diabetes | O24 | | N/A | | N/A | | N/A |
| Gestational hypertension | O13, O16 | | N/A | | N/A | | N/A |
| Cause of death |  | |  | |  | |  |
| All avoidable deaths (excluding cancer) | ICD-10 codes listed below for: unintentional injuries, intentional injuries, drug use disorders, and all other avoidable deaths | | N/A | | N/A | | N/A |
| Unintentional  injuries | V01-V99; W00-X59 | | N/A | | N/A | | N/A |
| Intentional  injuries | X60-X84, Y10-Y34; X85-Y09, U50.9; Y60-Y69, Y83-Y84 | | N/A | | N/A | | N/A |
| Drug use disorders | F10, G31.2, G62.1, I42.6, K29.2, K70, K73, K74 (excluding K74.3-K74.5), K86.0; F11-F16, F18-F19 | | N/A | | N/A | | N/A |
| All other  avoidable deaths | A15-A19, B90; A38-A41, A46, A48.1, B50-B54, G00-G03, J02, L03; B17.1, B18.2; B20-B24, E10-E14, G40-G41, I01-I09; I10-I15; I20-I25; I26, I80.1-I80.3, I80.9, I182.9; I160-I69; I70, J09-J11; J12-J18; J40-J44; J45-J46, K25-K28; K35-K38, K40-K46, K80-K83; K85, K86.1-K86.9, K91.5, N00-N07, N17-N19, N25-N27; N13, N20-N21, N35, N40, N99.1, P00-P96, A33; Q00-Q99 | | N/A | | N/A | | N/A |
| Cancer (avoidable and unavoidable) | C00–D48 | | N/A | | N/A | | N/A |
| All unavoidable deaths (excluding cancer) | All other ICD-10 cause of death codes not previously listed | | N/A | | N/A | | N/A |

Note. A diagnosis code ending with “.x” indicates that all codes that begin with the digits preceding the decimal were included, unless otherwise specified.

^a^ ICD- 10 = International Statistical Classification of Diseases and Related Health Problems, 10^th^ Revision

†ICD-9 = International Classification of Diseases and Related Health Problems, 9th Revision

‡ DSM-IV = Diagnostic and Statistical Manual of Mental Disorders, 4th Edition

§ Ontario procedure codes are from the Canadian Procedural Codes (CCP and CCI); England procedure codes are from the UK Office for Population Censuses and Surveys classification (OPCS), 4^th^ revision.
