# Supplementary material for: Long-term mortality in mothers of infants with neonatal abstinence syndrome: A population-based parallel-cohort study in England and Ontario, Canada
Source: PLoS Med. 2019 Nov 26;16(11):e1002974. doi: 10.1371/journal.pmed.1002974 (PMC6879118; doi:10.1371/journal.pmed.1002974)
Supplement: S2 Table — (DOCX) [file pmed.1002974.s002.docx]

**S2 Table.** Description of baseline sociodemographic characteristics for neighbourhood income quintile and urban and rural area of residence

| Baseline characteristics | England | Ontario |
| --- | --- | --- |
| Neighbourhood income  quintile | Quintiles of Income Deprivation Domain measured at Lower Super Output Area (LSOA) level across England. LSOAs are small areas of a similar population size, averaging approximately 1,500 residents or 650 households, based on the 2011 census. The Income Deprivation Domain measures the proportion of the population experiencing deprivation relating to low income (defined as people who are out-of-work, and those that are in work but who have low earnings that satisfy means tests). | Individuals’ postal codes were first matched to dissemination areas (DAs), the smallest available geographic census area, where the average income per single-person equivalent (weighing for household size) was obtained from the 2006 Canadian census. DAs within each metropolitan census area were ranked, and assigned to five groups, or quintiles, of approximately equal size. The corresponding neighbourhood income quintile of that DA was assigned to the individual. |
| Urban area of  residence | Urban output areas with >=10k population and the wider surrounding area is less sparsely populated. | Based on methods developed by Statistics Canada. As of 2010, the term ‘population centre’ replaced ‘urban area’. Population centres are considered an area with a population of at least 1,000 and a density of 400 or more people per square kilometre. |
| Rural area of  residence | More sparse areas with <=10k population. | All areas outside population centres are defined as rural area. |
