# Supplementary material for: Long-term mortality in mothers of infants with neonatal abstinence syndrome: A population-based parallel-cohort study in England and Ontario, Canada
Source: PLoS Med. 2019 Nov 26;16(11):e1002974. doi: 10.1371/journal.pmed.1002974 (PMC6879118; doi:10.1371/journal.pmed.1002974)
Supplement: S1 Text — (DOCX) [file pmed.1002974.s003.docx]

| Project Cohort | | |
| --- | --- | --- |
| **Study Design** | Cohort study  Matched cohort study  Case-control study  Cross-sectional study  Other (specify): | |
| **Index Event / Inclusion Criteria** | Live births in MOMBABY (April 1, 2002 <= B_BDATE <= DECEMBER 31, 2012)  **Cases (referred to as NAS mother) =** Mothers aged 12 to 49 years who gave birth to an infant with a diagnosis of i) neonatal abstinence syndrome (P96.1) or ii) newborn affected by maternal use of drugs of addiction (P04.4)  **Controls (referred to as non-NAS mother)** = Mothers aged 12 to 49 years who did not give birth to an infant diagnosed with P96.1 or P04.4  **Note**: For mothers who have more than one pregnancy during the study period, index date will be:   - **Cases:** date of the delivery resulting in the birth of an infant born with P96.1 or P04.4 (referred to as NAS infant). If a mother has more than one NAS infant, a random delivery date (i.e. B_BDATE) resulting in the birth of an NAS infant will be selected as index date. - **Controls:** a random delivery date will be chosen as index date. | |
| **Estimated Size of Cohort**  **(if known)** | Unknown | |
| **Exclusions (in order)** | *Step* | Description |
|  | 1 | Still births (b_stillbirth ne ‘F’ or m_stillbirth ne ‘F’)   - Flag records with stillbirth and exclude from index date selection |
|  | 2 | Invalid IKN (m_IKN or b_IKN) |
|  | 3 | WARN ne ‘N’ |
|  |  |  |

| Project Time Frame Definitions | | |
| --- | --- | --- |
| Look-back Window  Observation Window  (in which to look for outcomes)  **Index Event Date**  Accrual Window  Max Follow-up Date | |  |
| **Accrual Start/End Dates** | B_BDATE: April 1, 2002 to December 31, 2012 |  |
| **Max Follow-up Date** | All-cause mortality: March 31, 2016  Cause-specific mortality: December 31, 2014 |  |
| **When does observation window terminate?** | - Max follow-up date: March 31, 2016 - Death |  |
| **Lookback Window(s)** | - To 1997 (for time since birth) - 5 years (for chronic condition hospitalizations)   **Note**: for Previous addictions-related hospitalizations, please exclude index date when doing 5-year lookback |  |

| Variable Definitions (add additional rows as needed) | |  |
| --- | --- | --- |
| **Main Exposure or Risk Factor** | All hospital births between April 1, 2002 to December 31, 2012:   - Take all records from MOMBABY dataset with b_bdate from fiscal year 2002 to calendar year 2012 (MOMBABY will capture all hospital births) - Link the records with CIHI-DAD where b_IKN=IKN | |
|  | **NAS mothers**:   - Take all baby records with a diagnosis of **ICD-10 code P96.1 or P04.4** as any diagnosis type within the first 14 days of birth (AAP_NAS guidelines) - Use the variable identifier that links to the mom’s birth record in CIHI/DAD data (m_IKN) to obtain cohort of mothers who gave birth to infants with NAS | |
|  | **Non-NAS mothers**:   - All live births of infants without a P96.1 or P04.4 diagnosis in MOMBABY between April 1, 2002 and December 31, 2012. Mothers will be identified using the variable identifier that links to the mom’s birth record in CIHI/DAD data (m_IKN) - Mothers identified as cases will be excluded from this cohort   **Note**: Prior to identifying the control population, please identify the NAS mothers (cases) first, then exclude them from the total population. We want to ensure a NAS mom who also gave birth to a non-NAS baby will not be randomly selected as a control. | |
| **Primary Outcome Definition** | **Deaths**   - Dataset(s) used: RPDB, ORGD   **Cause of death will be categorized as:**   - Avoidable (excluding cancer) - Unavoidable (excluding cancer) - Cancer (avoidable + unavoidable)  1. **Avoidable deaths** (categories developed by the Office of National Statistics, UK) 2. Drug use disorders 3. Unintentional injuries 4. Intentional injuries 5. Other avoidable deaths (excl. cancer)  \| **Drug use disorders**: Alcohol related diseases excluding external causes; illicit drug use disorders \| F10, G31.2, G62.1, I42.6, K29.2, K70, K73, K74 (excluding K74.3-K74.5), K86.0; F11-F16, F18-F19 \| \| --- \| --- \| \| **Unintentional injuries**: Transport accidents; Accidental injury \| V01-V99; W00-X59 \| \| **Intentional injuries**: Suicide and self-inficted injuries; Homicide/Assault; Misadventures to patients during surgical and medical care \| X60-X84, Y10-Y34; X85-Y09, U50.9; Y60-Y69, Y83-Y84; ORGD variable MANNER_OF_DEATH = 4 (Ontario data only) \| \| **Other avoidable deaths (excl. cancer)** \| A15-A19, B90; A38-A41, A46, A48.1, B50-B54, G00-G03, J02, L03; B17.1, B18.2; B20-B24, E10-E14, G40-G41, I01-I09; I10-I15; I20-I25; I26, I80.1-I80.3, I80.9, I182.9; I160-I69; I70, J09-J11; J12-J18; J40-J44; J45-J46, K25-K28; K35-K38, K40-K46, K80-K83; K85, K86.1-K86.9, K91.5, N00-N07, N17-N19, N25-N27; N13, N20-N21, N35, N40, N99.1, P00-P96, A33; Q00-Q99 \|  1. **Unavoidable deaths**   All remaining deaths not identified above (excluding cancer) will be considered unavoidable deaths and categorized under a single ‘unavoidable deaths’ category.   1. **Cancer deaths (avoidable + unavoidable)**  \| **Neoplasms**: lip, oral cavity and pharynx; esophagus; stomach; colon and rectum; liver; trachea, bronchus and lung; skin; mesothelioma; breast; cervix uteri; bladder; thyroid gland; Hodgkin’s disease; Leukaemia; benign neoplasms \| C00-C14; C15; C16; C18-C21; C22; C33-34; C43; C45; C50; C53; C67; C73; C81; C91, C92.0, D10-D36 \| \| --- \| --- \| | |
| **Baseline Characteristics / Covariates** | 1. **Characteristics of the mother (no lookback period):**  - Age, years, mean (+ SD) - Age categorized as follows: - 12-19 - 20-34 - 35-49 - Neighbourhood income quintiles (RPDB; use %getdemo to extract INCQUINT) - Q1 (includes suppressed values), Q2, Q3, Q4, Q5, Q2-Q5 combined - Area of residence (RPDB; use %getdemo) - Urban - Rural - Obstetrical complications: - Caesarean delivery: **Procedure code 1-10 = 860, 861, 862; Procedure code 1-20 = 5MD60** - Preterm labour (DAD variable GESTWKS/GESTWKS_DEL for Ontario data only): - < 34 weeks - 34–36 weeks - 37+ weeks - Gestational hypertension: **ICD-10-CA code O13, O16** - Eclampsia: **ICD-10-CA codes O14, O15** - Gestational diabetes: **ICD-10-CA code O24;** - Infants discharged/detained by social services - Neonatal mortality (i.e. death within the first 28 days of life)  1. **Characteristics of the mother (includes lookback period):**  - Time since last birth (MOMBABY; DAD); lookback to 1997 - No previous births since 1997 - < 2 years - 2 to 5 years - 6+ years - Charlson comorbidity index; 5-year lookback - Mean (+ SD); Categories: 0, 1, 2+ - History of psychiatric hospitalizations (see Appendix A for diagnostic codes); 5-year lookback - Any psychiatric condition - Addictions-related - Other mental health (excludes addictions-related) | |
| **Statistical Analysis** | - **Table 1: Characteristics of mothers at baseline, April 2002 to December 2012** - Baseline characteristics will be compared between cases and controls using a Pearson’s chi square for categorical variables and ANOVA or Kruskal-Wallis tests for continuous variables - **Table 2: Bivariate age-standardized all-cause mortality rates per 1000 women, April 2002 to March 2016 (age-standardized to the 2006 Canadian population)** - Maternal death post-delivery - Overall; 5-year mortality; 10-year mortality - Maternal Age at delivery - ≤ 19, 20–34, 35+ - Neighbourhood income quintile - Q1 (includes missing income); Q2–Q5 combined - Area of residence - Rural; Urban (includes missing) - Previous addictions-related hospitalization (5 years, excluding index date) - Yes; No - Previous any other mental health hospitalization (5 years, including index date) - Yes; No - Infant discharged to social services - Yes; No - **Table 3: 10-year cumulative risk, cause-specific mortality per 1000 women for NAS mothers vs. non-NAS mothers, April 2002 to December 31, 2014** - Avoidable - Unintentional; Intentional; Drug use disorders; All other avoidable - Unavoidable - Cancer - Missing - **Table 4: All-cause (overall) mortality rates using Cox regression, with proportionality of hazards assesssed; the following models in this order:** - NAS - NAS, Maternal age at first delivery - NAS, Maternal age at first delivery, Charlson - **Figure 1: Kaplan-Meier curves crude and adjusted for Maternal age at delivery and Charlson** - **Supplementary analyses:**   **Cox models including additional factors, in the following order:**   - NAS - NAS, Maternal age at delivery - Previous model + Charlson | |
| **Additional analyses after reviewer comments** | - **Percentage of mothers (cases vs. controls) whose infant from index delivery date had:** - P96.1 record (P96.1 only or P96.1 + P04.4) - P04.4 only record - **Table 3. Age-standardized all-cause mortality rates per 1000 women, 2002 to 2016** - Produce mortality rates stratified by Charlson comorbidity index categories: 0, 1+ - **Figure 1. Survival curves for all-cause mortality (Kaplan-Meier curve)** - Re-produce adjusted curve without Charlson (i.e. only adjust for maternal age at first delivery) | |

**Appendix A. List of psychiatric hospitalization diagnostic codes**

|  | **International Statistical Classification of Diseases and Related Health Problems, 10^th^ Revision** | **International Statistical Classification of Diseases and Related Health Problems, 9th Revision** | **Diagnostic and Statistical Manual of Mental Disorders, 4th Edition (DSM-IV)*** |
| --- | --- | --- | --- |
| Any psychiatric condition | F04 to F99, G24.0, G31.2, G40.5, G62.1, G72.0, G72.1, I42.6, K29.2, K70, K85.2, K85.3, K86.0, O35.4, R78.1-R78.5, Y47, Y49, Z50.2, Z50.3, Z71.4, Z71.5, Z72.2, Z86.4, Z09.3, Z50.4, Z86.5, Z91.4, X60-X84, Y10-Y19, Y28, Y87.2, Y34 | 290-319, V673, V5789, V118, V1549, 3337, 30390, 3457, 3575, 3594, 3594, 4255, 53530, 5710, 5770, 5770, 5771, 65541, 7960, 7960, 7960, 7960, 7960, E9370, E9390, V5789, V5789, V6542, V6542, V698, V1589, 5711, 5712, 5713, 5728, 65543, 65540, E9394, E9371, E9372, E9373, E9376, E9378, E9379, E9390, E9391, E9392, E9393, E9396, E9397, E9398, E9399, E950-E958, E980, E986 | All DSM-IV codes, excluding 290.x and 294.x |
| Addictions-related | F55, F10 to F19, G24.0, G31.2, G40.5, G62.1, G72.0, G72.1, I42.6, K29.2, K70, K85.2, K85.3, K86.0, O35.4, R78.1-R78.5, Y47, Y49, Z50.2, Z50.3, Z71.4, Z71.5, Z72.2, Z86.4 | 291.x (0-5, 8, 9), 292.x (0, 1, 2, 8, 9), 303.x, 304.x, 305.x, 3337, 30390, 3457, 3575, 3594, 3594, 4255, 53530, 5710, 5770, 5770, 5771, 65541, 7960, 7960, 7960, 7960, 7960, E9370, E9390, V5789, V5789, V6542, V6542, V698, V1589, 5711, 5712, 5713, 5728, 65543, 65540, E9394, E9371, E9372, E9373, E9376, E9378, E9379, E9390, E9391, E9392, E9393, E9396, E9397, E9398, E9399 | 291.x (excluding 291.82), 292.x (excluding 292.85), 303.x, 304.x, 305.x |
| Other mental health (excludes addictions) | F04 to F99, Z09.3, Z50.4, Z86.5, Z91.4,  X60-X84, Y10-Y19, Y28, Y87.2, Y34; excludes above addictions-related codes | 290-319, V673, V5789, V118, V1549, E950-E958, E980, E986’ excludes 290.x, 294.x, and above addictions-related codes | All DSM-IV codes, excluding 290.x, 294.x, and above addictions-related codes |

***DSM-IV diagnostic codes were only used for the Ontario hospital data. Ontario hospital data also included ICD-10 and ICD-9 codes.**

**Ontario to English data mapping:**

The analytic plan outlined (above) was applied to a parallel cohort of NAS and control mothers identified using English hospital and mortality data.

**English Datasets:**

**Health data:** De-identified Hospital Episode Statistics Admitted Patient Care data (1 Apr 1997-31 Dec 2012) for women aged 12-49 years, with mother-baby link (Apr 2002-Dec 2012) derived using methods by Harron et al. 2016

**Mortality data:** Linked Office for National Statistics mortality data (1 Apr 2002-31 Mar 2016)

All definitions were as closely aligned as possible (i.e. the same ICD 10 codes, clinical groupings and/or time specfications were applied) between Ontario and English data. Differences in variable definitions are as follows:

**Variable definitions (where these differ to Ontario variables):**

Neighbourhood income quintiles (IMD04)

Area of residence (RURURB_IND with 5 indicates urban areas)

Caesarean delivery (Procedure code R17, R18 and/or DELMETH =7 or 8)

Preterm labour (GESTAT with same week groupings as Ontario data)

Gestational hypertension: ICD-10 codes: O13, O16

Eclampsia: ICD-10 codes: O14, O15

Gestational diabetes: ICD-10 code O24

Infants discharged/detained by social services (DISDEST specified as LA care / LA foster care in infant record)

**Linkage methods**

Harron K, Gilbert R, Cromwell D, van der Meulen J, Linking Data for Mothers and Babies in De-Identified Electronic Health Data*. PLoS One* 2016:11; e0164667
