# Supplementary material for: Long-term mortality in mothers of infants with neonatal abstinence syndrome: A population-based parallel-cohort study in England and Ontario, Canada
Source: PLoS Med. 2019 Nov 26;16(11):e1002974. doi: 10.1371/journal.pmed.1002974 (PMC6879118; doi:10.1371/journal.pmed.1002974)
Supplement: Attachment — Submitted filename: 00_Response to Reviewers_PLOS Med NAS Paper_FINAL.docx [file pmed.1002974.s005.docx]

September 17, 2019

Dear Dr. Stone,

Thank you for your continued consideration of our manuscript “Long-term mortality in mothers of infants with neonatal abstinence syndrome: A population-based parallel-cohort study in England and Ontario, Canada”. We enclose our responses to comments from the editors and reviewers below and think that we have been able to address all of the comments. In particular, we have clarified that our intent was not to make causal inferences so we have removed the comorbidity measure from the adjusted Cox proportional hazards models and have noted this change from the planned analyses in the manuscript.

We would be pleased if this manuscript were included the Special Issue on Substance Use, Misuse and Dependence.

Sincerely,

Astrid Guttmann MDCM, MSc FRCPC

Senior Scientist and Chief Science Officer, ICES

| **Manuscript section** | **Reviewer comments** | **Author’s response** |
| --- | --- | --- |
| ***Requests from the editors*** | | |
| **Title** | Please revise your title according to PLOS Medicine's style. ‘Findings from’ can be omitted. | ‘Findings from’ has been removed from title. |
| **Abstract** | Please remove the hyphens between opioid and addiction / use. | Hyphens have been removed. |
|  | Please add summary demographic details for study participants early in the "methods and findings" subsection of your abstract. | Summary demographic details were moved up to the second sentence of the Methods and Findings section of the Abstract. |
|  | Please clarify why death records only extended to 10 years after delivery when health data was assessed from 2002 and death records assessed up until 2016 | “up to 10 years” has been removed from the sentence as all deaths were captured in our analyses. |
|  | Please ensure that all numbers presented in the abstract are present and identical to numbers presented in the main manuscript text | The summary demographic details for the control study participants (i.e. n counts) have been added to the first sentence of the Cohort Characteristics section of the Results section of the manuscript text. |
|  | Please clarify what is meant by ‘index delivery’ | The term ‘index delivery’ has been removed and replaced with ‘date of delivery’. |
|  | Please include 95% CIs and p values for 10-year mortality data. | We have added 95% Cis and p-values for the 10 year mortality rates. |
|  | Please clarify which of the data presented are observed or modelled | This has been clarified by revising the following sentence: “10-year mortality was 5.1% …” to “Estimated 10-year mortality based on Kaplan-Meier curves was 5.1% …” |
|  | Please include the important dependent variables that are adjusted for in the analyses | We have added that we adjusted for maternal age at delivery to the Methods and Findings section of the Abstract. |
|  | Please expand upon what was considered an ‘avoidable cause’ of death | We have expanded the explanation with “defined as deaths that were either preventable, amenable to care, or both” in the Methods and Findings section of the Abstract. |
|  | Please add the main finding from the control group for comparison. You state in the first line of your discussion that ‘a mortality risk that is 11-12 times higher than for control mothers’, for example. | The main finding for the control group has been added. |
|  | Please interpret the study based on the results presented in the abstract, emphasizing what is new without overstating your conclusions. | We have reiterated the finding of a mortality risk 11–12 times higher than for control mothers (to justify our concluding sentence around the importance for policy responses) and added to the conclusions a sentence contextualizing the finding around the survival curves showing no infection point in the current practice limiting public health programs such as nurse home visits to the early postpartum period so as to anchor the recommendation in the final sentence around “long-term” support in our findings. We have also added “Survival curves showed no clear inflection point or period of heightened risk” to the abstract findings.  “In this study, we found that approximately one in 20 mothers of infants with NAS died within 10 years of delivery in both England and Canada— a mortality risk 11–12 times higher than for control mothers. Risk of death was not limited to the early postpartum period targeted by most public health programs. Policy responses to the current opioid epidemic require effective strategies for long-term support to improve the health and welfare of opioid using mothers and their children.” |
|  | Please begin the first sentence of the "conclusions" subsection of your abstract with "In this study, we found that ..." or similar. | ‘In this study, we found that…’ has been added. |
| **Author summary** | At this stage, we ask that you include a short, non-technical Author Summary of your research to make findings accessible to a wide audience that includes both scientists and non-scientists. The Author Summary should immediately follow the Abstract in your revised manuscript. This text is subject to editorial change and should be distinct from the scientific abstract. Please see our author guidelines for more information: <https://journals.plos.org/plosmedicine/s/revising-your-manuscript#loc-author-summary> | We have included this new section |
| **Introduction** | Please explain the need for and potential importance of your study. | We have added more justification to the Introduction in 2 sections:  “Pregnancy and delivery care are opportunities to improve access to addiction and supportive services. While public health programs such as nurse home visits tend to focus on pregnancy and the early post-partum period [15], it is largely unknown whether this is the only period of risk for poor outcomes for mothers who use opioids in pregnancy.”  “There is a dearth of information about the long-term health outcomes of women – particularly mothers – with opioid use, which is an important omission given rising rates of prescription and illicit opioid use, pregnancy as a window of opportunity for identifying and managing substance misuse and the implications for parenting capacity.” |
|  | If there has been a systematic review of the evidence related to your study (or you have conducted one), please refer to and reference that review and indicate whether it supports the need for your study. | There is no systematic review available which is why we included in a table our evidence review which highlights the dearth of data on maternal mortality related to opioid use |
|  | Please conclude the Introduction with a clear description of the study question or hypothesis, including what is measured as primary and secondary outcomes | Our objectives (as outlined in the original study protocol) were to:  1. Describe outcomes of death: all-cause mortality (primary outcome) and cause-specific mortality (secondary outcomes) for mothers of infants born with neonatal abstinence syndrome (NAS).  2. Compare study outcomes and baseline characteristics for mothers of infants born with NAS to a control population of mothers of infants born without NAS  We have now refined our introduction to make these objectives explicit and these were also outlined in the analytic/data set creation plan that we have added as an Appendix (see below). |
| **Methods** | Did your study have a prospective protocol or analysis plan? Please state this (either way) early in the Methods section. If available, please include the relevant prospectively written document with your revised manuscript as a Supporting Information file to be published alongside your study, and cite it in the Methods section. A legend for this file should be included at the end of your manuscript. If no such document exists, please make sure that the Methods section transparently describes when analyses were planned, and when/why any data-driven changes to analyses took place. In either case, changes in the analysis-- including those made in response to peer review comments-- should be identified as such in the Methods section of the paper, with rationale. | We have included a copy of the study’s analytic plan in this revised submission. We have added to the Methods section:  “ An analytic plan was written and approved before starting statistical analyses (S1 Dataset Creation Plan). All analyses were undertaken as planned and reported, with the exception of the removal of comorbidity adjustment in the Cox proportional hazards model (see section on Statistical Analysis) as a result of feedback from peer review. |
|  | Please indicate: (1) the specific hypotheses you intended to test, (2) the analytical methods by which you planned to test them, (3) the analyses you actually performed, and (4) when reported analyses differ from those that were planned, transparent explanations for differences that affect the reliability of the study's results. | We have added these elements to the end of the Introduction, the first sentence of methods section, and to the Statistical analysis sections. |
|  | Please clarify what is meant by ‘Mothers and infants and these different datasets were linked’ | The quoted section has been removed and the sentence has been revised to read “Datasets were linked using unique coded identifiers common across all aforementioned datasets and analyzed at ICES in Toronto, Ontario, Canada.” |
|  | Please clarify what is meant by ‘randomly selected one delivery date’ | The sentence has been revised to be clearer.  “We restricted the cohort to singleton births and if a woman had more than one livebirth delivery during the study period, a delivery date was selected at random and used as the study entry point for the mother (referred to as index delivery).” |
|  | Please clarify what is meant by ‘index delivery’ | The term ‘index delivery’ has been clarified (please see above). |
|  | Please ensure that the study is reported according to the STROBE guideline. Please add the following statement, or similar, to the Methods: "This study is reported as per the Strengthening the Reporting of Observational Studies in Epidemiology (STROBE) guideline (S1 Checklist).” | The study was reported according to the STROBE guideline and the suggested statement has been added to the Methods section. |
| **Results** | Where results are presented in the cohort characteristics, please quantify the differences between mothers of infants with NAS and controls with % and p values. (page 9) | We have done this throughout the section entitled “Cohort characteristics of mothers in England and Ontario” |
|  | On page 9, please clarify what you mean by "significant". If statistical significance is intended, please indicate that. | We have indicated where significance was meant to denote statistical significance |
|  | When a p value is given, please specify the statistical test used to determine it. | We have annotated Table 2 to associate each p value with the tests outlined in the Methods sections under the section Descriptive statistics |
|  | Please avoid general terms like ‘similar’ and ‘generally’ | We have removed these terms. For the manuscript section related to Table 2 we had used these terms predominantly to make inferences across jurisdictions but since we did not test these differences we have now omitted any descriptions of results that suggested comparison between mortality rates of subjects in England and Ontario. For our descriptions of differences in age-standardized mortality rates in Table 3 we have also added more precise language around which differences between NAS mothers and controls were significantly different (using overlapping confidence intervals).  “Differences in rates were not significant (95% confidence intervals overlapped) for NAS mothers and controls for those with a history of addictions-related admissions (England and Ontario), or who had an infant discharged to social services (Ontario only) or were under nineteen years at delivery (Ontario only).” |
|  | Please include 95% CIs and p values for 5-year and 10-year mortality data. | These have been added.  “In England, 5- and 10-year mortality with 95% CIs was 1.81% (1.59 -2.05%) and 5.13% (4.69-5.62%) respectively for mothers of infants with NAS, and 0.15% (0.15-0.16%) and 0.42% (0.41-0.43%) respectively for controls; while, for Ontario it was 1.85% (1.51-2.28%) and 4.58% (3.81-5.49%) respectively for mothers of infants with NAS and 0.15% (0.15-0.16%) and 0.40% (0.38-0.41%) respectively for controls.”  We also added the methods used:  “We derived 95% confidence intervals for mortality at 5 and 10 years after delivery through loglog transformation of the survival function and computed p values using the z test.” |
|  | Please indicate which factors are adjusted for in the main text (page 12) | We have clarified the text to indicate that the results presented on p12 are unadjusted. |
|  | The Supporting Information file Table S3 is central to the understanding of the paper. Please incorporate it into the main paper (page 13). | Table S3 has now been incorporated into the main paper as Table 4. |
|  | Please clarify why data not available up to 2016 (page 14). | We have reiterated from the Methods section that cause of death data were not available for Ontario beyond 2014.  “Table 5 presents results for cause-specific mortality between 2002 and 2014 (cause-specific data for 2014-16 were not available in Ontario).” |
|  | Please highlight in your results section the data for which you infer that ‘We also identified universally high mortality rates among mothers who had a history of hospitalization for mental health or addictions, irrespective of whether or not their infant had NAS.’ in your discussion. | We have now added further detail to, and clarified this section of the results:  “ In both jurisdictions, rates of death were higher among mothers of infants with NAS compared to controls across most risk groups. Differences in rates were not significant (95% confidence intervals overlapped) for NAS mothers and controls for those with a history of addictions-related admissions (England and Ontario)”  We edited the sentence in the discussion to include only addiction-related hospitalizations. |
| **Discussion** | Please present and organize the Discussion as follows: a short, clear summary of the article's findings; what the study adds to existing research and where and why the results may differ from previous research; strengths and limitations of the study; implications and next steps for research, clinical practice, and/or public policy; one-paragraph conclusion. | We have restructured the Discussion to follow this organization and highlighted in the first sentence of the second last paragraph that this was around implications and that the final one was in conclusion. We have also added discussion on how our findings fit in with other research on mortality rates in non-pregnant women using opioids as suggested by reviewer 1. |
|  | Please clarify what is considered a ‘ early high risk period’ (p.16) | We have added more detail:  “We found no evidence of high risk period in the 1–2 years after birth (corresponding to the period typically targeted by public health nursing or other support for high risk families)” |
|  | PLOS does not permit "data not shown” (page 17). Please remove this claim, or do one of the following: a) If you are the owner of the data relevant to this claim, please provide the data in accordance with the PLOS data policy, and update your Data Availability Statement as needed. b) If the data not shown refer to a study from another group that has not been published, please cite personal communication in your manuscript text (it should not be included in the reference section). Please provide the name of the individual, the affiliation, and date of communication. The individual must provide PLOS Medicine written permission to be named for this purpose. c) For any other circumstance, please contact me ASAP. | We have now removed this statement and replaced it with a reference (DOI <https://doi.org/10.23889/ijpds.v3i4.895>). |
| **References** | In your reference list, please ensure that journal names are abbreviated consistently, and add spaces where needed (e.g. reference 32 "BrMedBull"). | We have done so |
| **Tables** | Table 2 - please indicate which differences between NAS cases and controls are statistically significant (or are not, if this is easier) for ease of interpretation. The table legend is lengthy and some text could be replaced by * and NS annotations within the table itself. | We have done so |
| **Figures** | In Figure 1, please provide 95% Cis, and please indicate whether differences between 5-year mortality are statistically significant. | We have done so and have described the methods used (please see above). |
| ***Comments from the reviewers: Reviewer #1*** | | |
| **Introduction** | On page 4 of introduction (no line numbers) it is stated that "risks may differ given patterns of opioid use in the current global epidemic" but I don't think the patterns of use is tested within this study, and I don't believe that there is evidence within report that the effect of opioid use would differ depending on the population prevalence/patterns being assessed as opposed to the effects on an individual. | We did not mean to imply that we were directly testing the relationship of patterns of opioid use with mortality so we have now changed   - “Very high rates of longer-term maternal mortality have been reported in two older studies (one Australian and one Finnish) [18, 19], but these risks may differ given patterns of opioid use in the current global epidemic.”   To   - “Very high rates of longer-term maternal mortality have been reported in two older studies (one Australian and one Finnish) [18, 19], but these studies may not reflect the current opioid epidemic in North America or the UK [4].” |
|  | The study population of mothers seems to be relevant only in terms of the children providing a proxy measure of the opioid status, and that as stated on page 10 there was "no difference in neonatal mortality between infants with NAS and controls". As such, it may be relevant to expand further on how comparable the results from this study are in terms of mortality rates to other  studies that exist and have taken a more general population but with different issues of accurately recording opioid use as suggested in introduction (eg lack of prescription data, measuring illicit use). | The selection of a population of mothers reflects several considerations, including the opportunity to provide interventions to better support family units and reduce the need for out of home care placements – this is now clarified in the introduction (please see earlier comments on study rationale). In addition, in line with this valuable suggestion, we have provided further context from the literature of rates in non-pregnant women (Aldridge 2018) to the introduction and expanded further on these studies in the discussion.  “Notably, three largescale studies present crude mortality rates per 1000 person years of 6.5 (95% confidence interval 6.1-6.9) in New South Wales [ref Degenhardt L, 2009], ranged between 7.5 and 13.9 in opioid using women aged 15-44 years in California [ref Veldhuizen S, 2014 ], and 12.2 (10.3-14.4) and 19.7 (15-25.8) respectively, for female users of heroin or other opioids in a Danish study [ref Arendt M,  2011]. Our crude mortality rates per 1000 person years for mothers with infants with NAS of 5.01 (4.62-5.44) in England and 4.28 (3.63-5.02) in Ontario are comparatively lower, which is likely driven in part by a relatively younger age distribution in our study population.  For example, in the New South Wales study, Degenhardt et al. included all ages and reported increasing mortality rates with older age.” |
| **Methods** | Further detail is in my opinion necessary to justify the non-consideration of other covariates in the statement of statistical analysis section that "..other covariates may be on the causal pathway". While this could be a justification for excluding terms as adjustments in the model, there is no evidence within the report of the proposed directions of causality (eg in graph), and I would consider that some of the other available covariates (eg deprivation status) could potentially be a confounding factor as opposed to being caused in the first instance by opioid use. | We have now clarified in the introduction that the original study aims were to describe the excess mortality of the mothers of infants with NAS, relative to control mothers. We had never aimed to delineate causal pathways as our data come from only one time point and we would argue that even deprivation status could be on the causal pathway. We had originally adjusted for Charlson as we thought the majority of morbidities in the index were confounders, but as some reviewers pointed out this is not entirely true. Several reviewers have raised the issue of which, if any, covariates should be included in the model for all-cause mortality. As reflected in the range of reviewer comments and recommendations, there is no clear cut solution because the relationships between opioid-use in pregnancy and death are complex and are likely to co-vary over time.  After much discussion, we report mortality estimates that are adjusted for age only (in addition to our crude estimates). We have retained the table of unadjusted rates presented within strata of factors likely to be related to opioid use and death – e.g. psychiatric hospitalizations as these can be used to identify groups of women at higher risk of mortality |
|  | Given the categorisation of age and charlson score in some of the descriptive tables, it would be useful to confirm if in fact that continuous measures are used for the models that have been fitted as this could provide a more appropriate model than if they have been categorised in a relatively arbitrary way. | Our models adjust for the age bands outlined in table 2 (we no longer report estimates that adjust for Charlson index). We have also removed the mean Charlson score from Table 2. |
|  | Individuals would technically not be "censored at death" as for all cause-mortality death is the outcome/event of interest and there would be no time after death to censor (as there would be with the end of follow up) | Sentence has been revised and “censored at death” has been removed. |
|  | To asses rates by factors not in the models stratified analysis has been done, but this is without first having tested interaction terms of the terms under consideration and opioid group which would provide more robust interpretation of whether there are any genuine sub-group differences. | We have added further explanation into the introduction and methods explaining that our purpose in reporting stratum specific rates is exploratory/hypothesis generating and relevant to service provision for the identification of mothers at high risk for poor outcomes/mortality.  “We present all-cause mortality in relation to some key maternal characteristics at the birth episode, to identify clinically useful sub-groups with poor prognostic outcomes and guide opioid misuse policy and research.” |
| **Results** | In figure 1 results from adjusted models are shown in Kaplan Meier plots, but it is unclear exactly what results this is showing. Potentially it is for the average age and average charlson score across the entire study, and if so this could be made clear within the figure caption. | The adjusted plot is derived from the Cox model (as presented in the main analysis – i.e. adjusted for age (as a categorical variable). We have now updated the figure caption to reflect this. |
|  | The grouping of Q2-Q5 for neighborhood income quarintile in table 3 seems somewhat arbitrary instead of using the quintiles directly, particularly as missing data has been recoded into the lowest level which could also have an effect on how this comparison would be interpreted. Although the assumptions for recoding the missing income-quintile or rural/urban in general seems reasonable, the robustness of the results to these assumptions and whether there is any systematic reasons for why there would be missing data if not just random would be useful to comment on. | We now state that grouping of quintiles of deprivation was necessary in Table 3 due to a relatively low numbers of death for women in more affluent quintiles, which was problematic for age standardising.  The level of missing data on income quintile and area was very low and unlikely to have any bearing on how our results are interpreted. However, we agree that further explanation is useful here, and have now added further detail to the methods section (in Ontario the missing data are in fact suppressed and make the choice of addition to lowest quintile and urban defensible). Data on neighbourhood income quintile was missing/suppressed for 0.82% of cases and 0.57% of controls in England and 2.1% for cases and 0.4% controls in Ontario. Are of rural/urban residence was missing for 1.1% of cases and 0.54% of controls in England and 0.08% for cases and 0.02% for controls in Ontario. |
| **Discussion** | In the discussion section on page 16, it is noted that there is "potential for impact to  improve maternal and child outcomes", and similarly on page 18 "potential implications for their children",  but within the analysis done as part of this article it is only stated that there is no difference in mortality between NAS and control children in terms of mortality and no other child outcomes were considered or summaries shown. Based on the data from this study alone the statements on child outcomes are therefore not supported. However, there are also summaries in the discussion section relating to children not presented in the main results of the study ("..10-15% NAS affected infants do not return home with mother..",  "greater risk of placement in out of home care")  suggesting that there potentially is data available that could be shown to support the statement and give greater clarity on the outcomes for children. | We included the neonatal mortality only as a baseline characteristic so readers could compare these study populations to their own. We did not use this measure as a proxy for longer term child outcomes and do not have data on subsequent child welfare decisions (either return to mother or subsequent apprehensions). As we outline in the concluding paragraph we explicitly wanted to focus on the maternal outcomes as so few studies have done so.    We have further highlighted the findings around differences in discharge to social services in infants with NAS in the Results section describing Table 3 as reference point for the 10-15% in the Discussion:  “Compared to controls, infants discharged to care by social services was much more common among those with NAS in both England (9.7% vs. 0.1%, *p* < 0.001) and Ontario (15.2% vs. 0.1%, *p* *<* 0.001)”  We also added “arguably” to the first sentence of the second last paragraph of the Discussion to clarify that this is not from a measureable child outcome from our study:  “Our study has implications for research, practice and policy to improve maternal and arguably child outcomes related to prenatal opioid use.”    We have also deleted “and the potential implications for their children” from the end of the second sentence of the concluding paragraph:  “ The findings provide a stark reminder of the vulnerability and sustained poor outcomes of these mothers.” |
| ***Reviewer #2*** | | |
|  | I was a little confused about the study period and the look-back time, when reading through the first time. Was there linked hospital data back to 1 April 1997 for all? Was the look-back a consistent 5 years for all, or was it back to 1997 for all? All-cause mortality had data up to March 31 2016, so only those from 2002 to 2006 had a full 10 years of follow-up, is that correct? Was 10 years the maximum follow-up time for each individual? Please add further details in the methods. Please specify the average follow-up time per person (split by jurisdiction as well as NAS and controls) in the Results section. | We have clarified in the Methods section that there was a 5 year lookback for all mothers for previous psychiatric hospitalizations and for the calculation of the Charlson comorbidity score but that for previous births we went as far back as data were available (April 1 1997).  We clarified that for the overall survival analysis all deaths were included (not only until 10 years). We chose to report 5 and 10 year mortality as summary and interpretable measures.  We have added to the Methods section:  “ Survival analysis for time to all-cause mortality was modelled using multivariable Cox regression, with proportionality of hazards assessed by Schoenfeld residuals and log-log plots and included all deaths to the end of the study period.”  “Individuals surviving beyond the end of the study period were censored at March 31, 2016”  We have also added the person-years of follow up to the results.  “The mean duration of follow-up was 8.4 years (England) and 7.2 (Ontario) for NAS mothers and 8.2 years (England) and 8.6 (Ontario) for controls.” |
|  | The abstract background discusses the pregnancy and delivery settings as an opportunity to access support, and potentially assist new mothers with opioid addiction. However, I couldn't see mention of this in the Introduction. If this is an important theme of the paper, then I think it needs further exploration. What, if any, studies have shown an impact of targeting pregnant women or mothers with opioid addiction? Are women on methadone or other opioid-replacement therapy better off than those who are not? | We have added additional information to the introduction to make the importance of pregnancy as a window of opportunity for intervention and support, explicit.  “There is a dearth of information about the long-term health outcomes of women – particularly mothers – with opioid use, which is an important omission given rising rates of prescription and illicit opioid use, pregnancy as a window of opportunity for identifying and managing substance misuse and the implications for parenting capacity. “  As we are not able to measure the type of opioids used or the type of supports received (and have mentioned these as important limitations) and have clarified that our aims were not around causal inferences, we have kept the discussion and references on programs targeting pregnant women predominantly in the Discussion section around implications for research, practice and policy |
|  | Please state the number and proportion of all the missing data noted in the methods, e.g. income, area of residence and age. The authors state that missing values for neighbourhood income quintile were put in the lowest income quintile, and that missing area of residence was categorised into urban. Including the missing data in one category will bias your findings towards the null, as the missing presumably include a proportion of values in all categories. I would suggest either removing the missing, if the proportion is very small, or using principled missing data methods such as multiple imputation or full information maximum likelihood, if the missing is more substantial. And why is the missing data for gestational week of birth so high in the English cohort? | Please see the earlier comment to reviewer 1 regarding the very low levels of missing data on income and area of residence and the justification for combining the missing data with lowest income quintile and urban.  The proportion of missing data on gestational age in the English data is in line with other studies using hospital episodes statistics data. This is because data on gestational age derived from the maternity records is not a mandated return to NHS Digital, thus data completeness is lower than other diagnostic or procedural codes. We have added a explanatory note to the legend of Table 2.  Ref : Annie Herbert, Linda Wijlaars, Ania Zylbersztejn, David Cromwell, Pia Hardelid, Data Resource Profile: Hospital Episode Statistics Admitted Patient Care (HES APC), International Journal of Epidemiology, Volume 46, Issue 4, August 2017, Pages 1093–1093i, https://doi.org/10.1093/ije/dyx015 |
|  | The Charlson Comorbidity index is a weighted score based on the relative risk of mortality. The index is not a continuous variable, nor a count of comorbidities (please revise the footnote on Table 2). Using it as a continuous variable (e.g. reporting mean and including it in the Cox regression as a continuous variable) suggests that the relative impact on mortality from 0 to 1 comorbidity score is the same as that from 4 to 5, for example. What is the justification for using it as a continuous variable? Has the functional form been tested and shown to be linear? | Please see our earlier comments on covariate adjustment -- our final models no longer adjust for Charlson index and we have taken the median value out of Table 2. |
|  | I agree that it is important to control for other underlying causes of death, not related to the possible opioid addiction, but there are some aspects of the Charlson score that are probably on the causal pathway, like HIV/AIDS and liver disease. I think a directed acyclic graph of the theoretical relationships between the variables and the mortality outcome would make the assumptions about the causal pathways explicit. | Please see our earlier comments on covariate adjustment and our clarification around the main aims of this study. |
|  | Page 7, para 3, line 7: Suggest removing "young", as the extreme values are young and old. Page 7, para 3, line 8: "Missing values for neighbourhood income quintile" should be followed by "were" not "was". Page 12, para 1, line 3: I understand the intent of this sentence but on first read, it sounds like the cumulative mortality was similar for those with NAS and controls. Suggest rewording. | Thank you for the suggestions. These edits have been incorporated into the manuscript. |
|  | Table 2 - The percentage of primiparous women in the control groups looks very high. A quick look for other studies using Ontario or Toronto data, shows the proportion of primips is between 39 to 47%, not close to 60%. (E.g. Park et al, 2015, JOGC; Booth et al, 2017, CMAJ). I did not search for English studies. | There are a number of reasons why the women in our study will have higher rates of no previous pregnancies. In our study we randomly selected one birth for mothers who had more than one delivery during the study period, and report parity for mothers at the time of that delivery thus increasing the likelihood of including first pregnancies. As a result, the proportion of women who are primiparous will be higher in our cohort than in studies (such as your example by Park et al.) where all births in a given time period are included. In addition, given that we only have data on births back to 1997 and in the respective jurisdictions (so may miss births in mothers who migrate to either jurisdiction) . To clarify, we have removed the word primiparous from the Results section and replaced with “no previous recorded hospital births” (lookback to April 1, 1997) |
|  | Table 2 - I would be interested in whether the preterm births are planned or spontaneous. Is it possible to split this out? | While we agree that planned versus spontaneous preterm births would be interesting to assess, this breakdown goes beyond the scope of our paper and is not key to the outcomes explored in this study. |
|  | Page 17, para 2, line 2: What is the expected impact of these limitations on the mortality outcomes? | We have added a statement indicating that mortality for the NAS group is likely to be underestimated, and provide further explanation in the rest of the paragraph. |
|  | Page 18, para 1, line 6: Are there differences between England and Ontario really a source of 'bias', if the data are never combined? | Thank you for this comment and agree this is not a source of bias so we have removed this sentence from the limitations. |
| ***Reviewer #3*** | | |
|  | Table 2 would be strengthened by the addition of p-values to each variable. | These have now been added |
|  | Supplementary table 3 is so important. I would move it to the body of the paper. | We have now included this as Table 4 |
|  | Also Tables 3 and 4 would be strengthened by aOR. | Please see our earlier comments regarding covariate adjustment. |
| ***Reviewer #4*** |  |  |
| General Comments | A major concern is about the use and exclusion of covariates for adjusted models. If the point of the paper is to highlight a group of women with a very high mortality rate, women with infants born with NAS, then adjusted models may not be necessary. If the goal is to examine causality, then adjustment is needed, but then the choice to adjust only for Charlson index and maternal age at delivery becomes hard to defend because it may not be true that everything else is on the causal pathway. The exposure is having a child with NAS; it is hard to see that having a baby with NAS causes gestational age, pre-eclampsia, or urban-rural residence. If the exposure is just opioid use, then it might cause psychiatric admissions, but if admissions are a proxy for underlying mental health issues, the relationship could go the other direction. Certainly, income could go in either direction as well. If you are trying to look at causality, however, then would you want to create essentially equal comparison groups in all other factors besides having had an infant with NAS? Not controlling for other social circumstances of these mothers (such as income) creates comparison groups with inherently different mortality risks.  A conceptual model would help drive such analyses. What causal pathway do you hypothesize? Why is having a baby with NAS a predictor of maternal mortality? What is upstream, what is downstream, and what are confounders? Modeling with these concepts in mind could very well help with interpretation. For example, if social circumstances highly confound the observed associations, you might infer that something about them would be amenable to intervention. You are correct that social circumstances might also be on the pathway, but not adjusting for them leaves you with few clues to cause. Modern methods of mediation analysis may help you with disentangling. | Thank you for giving such helpful feedback, as a result we have clarified the objectives of the study, and the limitations of our data, in the introduction and methods and provided our reasoning for reporting crude and age-adjusted estimates of mortality only. |
|  | Page 2, para 2, line 4: NAS as imperfect proxy. Do data exist on sensitivity and specificity? Presumably specificity is high, sensitivity low. How might that alter your inferences? | We do not know of any studies that have calculated these and have described the issues with illicit use that make this a difficult endeavour. It is the reason however, that we also use P04.4 (see below). We have added an additional limitation that reflects on this:  “Limitations include linkage error, misclassification of mothers using opioids whose baby did not develop NAS, lack of direct measures of maternal opioid use or treatment, other substance misuse, which may underestimate the burden of mortality in mothers with opioid use within our study or make findings less generalizable to opioid using mothers whose infants do not have NAS” |
|  | Page 3, line 2: “Overall social disadvantage and opioid use are inextricably linked…” In what directions? Does that knowledge help you in modeling? | We further describe the complexity and time dependent nature causally linking socio-economic status, opioid use and death in the methods as a justification for our modelling strategy.  “We explicitly did not attempt to adjust models for other covariates as we do not attempt to infer causality. This is primarily because our data reflect maternal opioid use at a single time point (delivery). Thus we are unable to examine the direction of effect for key factors such as mental illness or socio-economic status that may either confound the association between opioid use and death, or lie on the causal pathway.” |
|  | Page 6: “randomly selected one delivery date…” Per woman? | Yes, we have clarified this in the Methods section |
|  | Page 6: The claim that ICD-10 P04.4 represents opioid exposed mothers is central to the paper. Hence, please include, perhaps in an appendix, the data that support the statement “…and correlated well with publicly funded prescriptions for opioids in a subsample of Ontario mothers who qualify for public prescription funding.” Also, in this respect, in identifying infants with NAS, the Agency for Healthcare Research and Quality (AHRQ) uses only P96.1, but not P04.4. Please consider how the results might differ if you used only P96.1, or at least what percentage of the cohort you identified using P04.4, and how much of a difference that could make. Finally, in this regard, I believe citation 30, which you used to justify the use of P96.1 actually looked at ICD-9 code 779.5, not ICD-10 P96.1. Consider citing something else or justify why they would be the same. | We have clarified that the citation used the ICD9 equivalent to P96.1 and we have added the AHRQ reference to the ICD10 code of P96.1 to the Methods as well as the the sensitivity -- 88.1% (95% CI: 83.3–91.7), specificity 97.0% (95% CI: 93.8–98.5) and positive predictive value of 91.2% (95% CI 86.8-94.2) of NAS to measure NAS.  We have also added more detail from new data not available when we initially submitted this manuscript to justify the use of P04.4:  “We included P04.4 as it is often used for opioid withdrawal in both jurisdictions. In a sample of all Ontario women giving birth in hospital from 2014-2017 during which time all prescription opioids were registered, over 56% of women whose infant had a diagnosis of P04.4 had either a prescription for opioids or opioid agonists, or a healthcare encounter related to opioid use during pregnancy”  We have also added the % of NAS by diagnosis code for both jurisdictions  “ In our study, NAS was ascertained by P96.1 in 83% of cases in England and 65.7% in Ontario.” |
|  | Page 7: The AHRQ Comorbidity Index (Elixhauser co-morbidity index) is a better co-morbidity index than Charlson at predicting mortality. The authors may want to consider using Elixhauser or justify their choice. | We have added justification for use of the Charlson-Deyo index which is the most commonly used Index in studies assessing maternal mortality. Unfortunately there are no comorbidity indices that have been specifically developed to predict longer term mortality in pregnant women or even younger women in general. While both the Elixhauser and Charlson index excluded pregnant women in the derivation samples, we chose the Charlson as it has been used most frequently for other studies of maternal mortality and was designed to predict 1 year mortality whereas the Elixhauser inpatient mortality. While Elixhauser has been shown to predict post discharge mortality, this has only been done in select cohorts. There is a new maternal co-morbidity index that has been published since we started this project but it too was validated to predict short term delivery outcomes and is not readily available.  In addition, with the changes to the modeling strategy, we are no longer adjusting for comorbidity. |
|  | Page7, top: Citation 31 that justifies the England mother/baby matching algorithm. Consider adding description of the performance of this matching algorithm. Did you do a sensitivity analysis to see how much your estimates would change at the extremes of assumptions. | We describe the proportion of infants with NAS who could not be linked to a mother, and have also added an additional citation for work investigating linkage bias within the study cohort but have not done a sensitivity analysis. |
|  | Page 7, 2nd paragraph under Outcomes and covariates, last sentence: Please report how much missing data exists for each of these variables, or at least all together. It is not clear why you would impute missing data the way you did. Other approaches would likely be superior, e.g., multiple imputation or, given the size of the data sets, perhaps a hotdeck or single imputation model based on the available other variables. | Please see our earlier comments regarding our approach to missing data |
|  | Page 7, third paragraph under Outcomes and covariates, first sentence: I may have missed this, but how can you have a time since last birth of 6+ years when the look-back period is only 5 years? | We have clarified that for all other baseline healthcare use we had a five year lookback window but for births we used a longer lookback period that was not fixed at 5 years |
|  | Page 8, All-cause mortality section, para 2: I think this is the first time the reader sees that you will examine associations of covariates with the outcomes. You may wish to foreshadow why in the introduction. | We have now added to the Introduction this aim:  “We present all-cause mortality in relation to some key maternal characteristics at the birth episode, to identify clinically useful sub-groups with poor prognostic outcomes and guiding opioid misuse policy and research.” |
|  | Page 16, para 1, last sentence: Do your data suggest that an early period (1-2 years) of family support does not work for this population? | We are unfortunately unable to address this question without data on the support that women in the study received but do not think the implication of the sentence is that these supports are ineffective.  We have added to the Limitations section:  “ Limitations include linkage error, misclassification of mothers using opioids whose baby did not develop NAS, lack of direct measures of maternal opioid use or treatment, other substance misuse, which may underestimate the burden of mortality in mothers with opioid use within our study or make findings less generalizable to opioid using mothers whose infants do not have NAS or live in other jurisdictions with other approaches to treatment and available supportive services” |
|  | Page 16, para 2: “studies and surveillance focused only on deaths directly attributable to opioid overdose will miss the full extent of the problem.” Please state why you reach this conclusion—you do not show data. | We have changed this sentence (which is now at the end of the second to last paragraph of the Discussion) to read:  “Finally, the findings from our study also indicate that studies and surveillance focused only on deaths directly attributable to opioid overdose will miss the full extent of the problem given the importance of deaths due to unintentional and intentional injuries, not all of which involve opioids.” |
|  | The figures are telling. You may wish to use a y-axis that starts at 0 instead of 0.8. That would show both that the curves are distinct and also more honestly the absolute rates. Also consider adding number of deaths and number at risk at the bottom, say at 5-year intervals. | As we are showing 4 graphs in this one exhibit we think starting at 0 will make the graph less easy to read unless the size of the exhibit is very large, however if the Editors feel strongly about this we can make the change. We have added number of deaths and those at risk to the figure note. |

Other important changes:

Table 2: The English numbers in Table 2 have been updated – in the corrected version the totals are 13,577 NAS mothers and 4,205,675 controls (as outlined in the abstract in text).
