# Supplementary material for: Long-term mortality in mothers of infants with neonatal abstinence syndrome: A population-based parallel-cohort study in England and Ontario, Canada
Source: PLoS Med. 2019 Nov 26;16(11):e1002974. doi: 10.1371/journal.pmed.1002974 (PMC6879118; doi:10.1371/journal.pmed.1002974)
Supplement: Attachment — Submitted filename: 00_Response to Reviewers2_PLOS Med NAS Paper_FINAL.docx [file pmed.1002974.s006.docx]

October 10, 2019

Dear Dr. Stone,

Thank you for your acceptance of our manuscript “Long-term mortality in mothers of infants with neonatal abstinence syndrome: A population-based parallel-cohort study in England and Ontario, Canada”. We enclose our responses to the minor comments from the editors and reviewers and thank you for a very smooth review process.

Sincerely,

Astrid Guttmann MDCM, MSc FRCPC

Senior Scientist and Chief Science Officer, ICES

| **Manuscript section** | **Reviewer comments** | **Author’s response** |
| --- | --- | --- |
| ***Requests from the editors*** | | |
| **Abstract and and text and tables** | Add p p values to all quantifiable data and where 95% Cis are provided | We have done this |
| **Author summary** | Remove the 2 bullets | We have omitted these |
|  | Clarify whether NAS mothers are more likely to be teenagers | In the Ontario data a higher proportion of teenaged vs older mothers have infants with NAS but we have removed this from the summary |
| **Discussion** | Add “to our knowledge” to page 21 “our study is the first population-based analysis” | We have added this disclaimer |
| **Data statement** | Point of contact cannot be an author | For both the English and Ontario sections, we have removed mention of access to the programming codes through the authors since we have included the dataset creation plan with all of the definitions that could be used to replicate the analyses. We have added more details on how data can be accessed to replicate the study and added contact information for Ontario |
| ***Comments from the reviewers: Reviewer #2*** | | |
|  | Clarify description of random selection of one birth per mother | We have edited this section with the recommended text |

Other changes:

We have added one more required disclaimer to the role of the sponsor/funder for the Ontario data
